# A reporter system for assaying influenza virus RNP functionality based on secreted *Gaussia *luciferase activity

**DOI:** 10.1186/1743-422X-8-29

**Published:** 2011-01-21

**Authors:** Wenfei Zhu, Jianfang Zhou, Kun Qin, Ning Du, Liqi Liu, Zaijiang Yu, Yun Zhu, Wenhong Tian, Xiaobing Wu, Yuelong Shu

**Affiliations:** 1 State Key Laboratory for Molecular Virology and Genetic Engineering, Chinese National Influenza Center, National Institute for Viral Disease Control and Prevention, China CDC, 155 Changbai Road, Beijing, 102206, PR China

## Abstract

**Background:**

Influenza A virus can infect a wide variety of animal species including humans, pigs, birds and other species. Viral ribonucleoprotein (vRNP) was involved in genome replication, transcription and host adaptation. Currently, firefly luciferase (Fluc) reporter system was used in vRNP functional assay. However, its limitation for the testing by virus infection resulted in an increased need for rapid, sensitive, and biosafe techniques. Here, an influenza A virus UTR-driven gene reporter for vRNP assay based on secreted *Gaussia *luciferase (Gluc) activity was evaluated.

**Results:**

By measuring Gluc levels in supernatants, reporter gene activity could be detected and quantitated after either reconstitution of influenza A virus polymerase complex or viral infection of 293T and A549 cells, respectively. As compared with Fluc reporter, Gluc-based reporter was heat-tolerant (65°C for 30 min) and produced 50-fold higher bioluminescent activity at 24 h posttransfection. Signals generated by Gluc reporter gene could be detected as early as 6 h post-infection and accumulated with time. Testing by viral infection, stronger signals were detected by Gluc reporter at a MOI of 0.001 than that of 1 and the effects of PB2-627K/E or amantadine on influenza vRNP activity were elucidated more effectively by the Gluc reporter system.

**Conclusions:**

This approach provided a rapid, sensitive, and biosafe assay of influenza vRNP function, particularly for the highly pathogenic avian influenza viruses.

## Background

Bioluminescence reporters such as *firefly*, click-beetle, *Renilla*, *Gaussia*, *Cypridina luciferases*, and calcium-activated photoproteins are widely used in molecular biology. Of them, the *firefly *luciferase (Fluc) from the North American *firefly*, first cloned and sequenced by Wet *et al*. in 1985, is the most commonly used [1]. *Gaussia *luciferase (Gluc), naturally secreted from the marine copepod, was reported by Tannous in 2005 and found to be ~2000-fold more sensitive than either the Fluc or *Renilla reniformis *luciferases [2]. Expression levels in growth medium are readily quantifiable by addition of coelenterazine and luminometry [2]. To date, Gluc has been used for the detection and quantification of protein interactions [3], siRNA [4], miRNA [5], promoter activity [6], and viral infection and replication [7]. Furthermore, the Gluc reporter system has also been used in live animal studies [8], and use of a bioluminescence detector [9] facilitated imaging of tumor cell trafficking and proliferation *in vivo*.

Influenza viruses are members of the family *Orthomyxoviridae *and are composed of three types: A, B, and C. They possess a single-stranded, negative-sense RNA genome consisting of eight (influenza A & B) or seven (influenza C) segments. Formation of a viral polymerase complex consisting of PB2, PB1, and PA proteins together with NP is required for successful replication [10]. Twelve conserved nucleotides at the 3', and 13 at the 5' UTR regions of vRNA are essential for polymerase recognition and binding for type A virus. Lutz and co-workers [11] introduced influenza A virus-inducible reporter gene segments (VIRGS, 3' and 5'UTR sequences of the A/WSN/33 NP segment) into a Fluc reporter system to detect and quantify RNP activity. This system has been used to study influenza virus RNP activity by transfection or viral infection of susceptible cells.

Co-transfecting cells with four plasmids encoding PB2, PB1, PA, and NP and a reporter gene avoids the generation of infectious virons and a BSL-3 facility for handling the highly pathogenic avian influenza virus. Moreover, RNP activity testing by viral infection of susceptible cells could be mandated when RNP expression plasmids are unavailable. However, a relatively weaker regulation mediated by virus infection directly than that by polymerase complex transfection limited the application of Fluc system. Therefore, it is necessary to develop a rapid, sensitive, quantitative, and safe assay for determining influenza virus polymerase activity. Here, we report a secreted influenza viral UTR-driven *Gaussia *luciferase (Gluc) reporter system for RNPs function assay.

## Materials and methods

### Plasmids

Plasmid pAAV-Gluc-Fluc (provided by Dr. Xiaobing Wu) was used as the template for Gluc reporter gene amplification. The primers used are listed in Table 1. After purification using the QIAquick Gel Extraction Kit (Qiagen, Valencia, CA, USA), PCR products were cloned into vectors containing the RNA polymerase I promoter and terminator using the In-Fusion(tm) Advantage PCR Cloning Kit (Clontech, Mountain View, CA, USA) according to the manufacturer's instructions. Plasmid of polI-Gluc was sequenced to ensure the absence of unwanted nucleotide changes.

**Table 1:** Primer sequences used in the construction of the viral UTR-driven Gluc reporter

Gluc-F	5'-*GGCCGCCGGGTTATT*AGTAGAAACAAGGGTATTTTTCTTTAGTCACCACCGGCCCCCTTGATC -3'
**Gluc-R1**	5'-*CCTCCGAAGTTGGGG*AGCAAAAGCAGGGTAGATAATCACTCACAGAGTGACATCGAAAAT-3 '

**Gluc-R2**	5'-CACAGAGTGACATCGAAA**ATGGGAGTCAAAGTTCTGTTTGCCCT**-3'

Bold portions correspond to sequences within the coding region of the reporter gene. The remaining 5' sequences (15 nt, italics) are necessary for In-Fusion cloning into the RNA polymerase I vector polI-*sapI*. Underlined parts correspond to the untranslated regions of the A/WSN/33 NP segment. Two reverse primers (R1, R2) were designed to ensure PCR efficiency.

All eight gene segments of influenza viruses A/Quail/HK/G1/97 (H9N2, G1) and BH Gs/QH/1/2005 (H5N1, QH) were amplified by RT-PCR and cloned into a modified version of the bidirectional expression plasmid pCQI, derived from pHW2K. A mutation resulting in a change of lysine (K) to glutamic acid (E) was introduced into the PB2 gene of QH strain; this virus was named QH (PB2-627E). The reporter plasmid pYH-Fluc was kindly provided by Erich Hoffmann.

### Cells

Human embryonic kidney (293T) and human type II alveolar epithelial (A549) cells were maintained in Dulbecco's modified Eagle's medium (DMEM; Invitrogen, Carlsbad, CA, USA) supplemented with 10% fetal bovine serum (FBS; Invitrogen), glutamine (2 mM; Invitrogen), HEPES (10 mM; Invitrogen), penicillin (100 units/ml), and streptomycin (100 μg/ml; Invitrogen) and incubated in a humidified atmosphere of 5% CO_2 _at 37°C.

### Virus preparation and titration

Recombinant viruses G1, QH (PB2-627K), and QH (PB2-627E) were maintained as reported previously [12,13]. Viral titrations were determined using A549 cells, and the tissue culture infectious dose affecting 50% of the cells (TCID_50_) was calculated using the Reed-Muench formula [14]. All experiments involving live H5N1 viruses were performed in a BSL-3 facility.

### Transfection and virus infection for RNP activity measurement

Reporter plasmids pYH-Fluc (0.1 μg) and polI-Gluc (0.1 μg) were co-transfected with expression plasmids encoding PB2, PB1, PA, and NP into 293T cells (1 × 10^5 ^and 5 × 10^4 ^/well in 24- and 96-well plates, respectively) using the PolyFect (Qiagen) reagent according to the manufacturer's instructions. Mock transfections were performed with either pYH-Fluc or polI-Gluc alone, as appropriate. For assay RNP activity by virus infection, A549 cells (1 × 10^5 ^/well) were transfected with polI-Gluc, again using the PolyFect reagent. After 6 h incubation at 37°C, cells were washed with phosphate buffered saline (PBS), and then the virus was introduced at a multiplicity of infection (m.o.i.) of either 1 or 0.001. Mock infections were performed using sterile culture medium.

Inhibition of influenza A virus infection by amantadine was assessed by incubating polI-Gluc- or pYH-Fluc-transfected A549 cells with 0.21 μM amantadine for 30 min prior to the addition of the virus. After incubation for 1 h at 37°C, supernatant was decanted, and cells were washed with PBS and then incubated at 37°C in DMEM containing amantadine.

### Luciferase assay

Gluc activity in supernatants (cell-free media; 20 μl) was analyzed using a Gluc assay Kit (New England Biolabs, Beverly, MA, USA). Transfected or infected cells were gently washed twice in PBS, followed by the addition of 200 μl 1× passive lysis buffer (Promega, Madison, WI, USA). Cell lysates were harvested and centrifuged for 5 min at 10,000 rpm, and Fluc activity in supernatants (cell lysates; 20 μl) was measured by addition of D-luciferin (100 μl; Promega) and by measurement of fluorescence intensity for 10 s using a Modulus Luminometer machine (Promega).

### Statistical analysis

All determinations were performed in triplicate and repeated three times. Data are expressed as means ± SEM. Statistical significance was determined using non-parametric tests and the GraphPad Prism 5 software package (GraphPad Software). A *P*-value < 0.05 was deemed to indicate statistical significance.

## Results

### Efficacy of the Gluc reporter system

**Figure 1:**
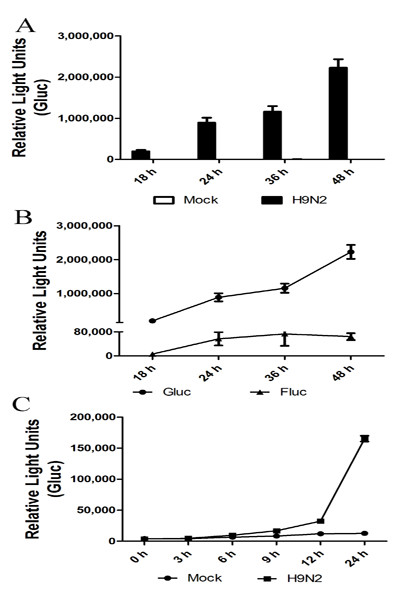
**RNP assaying by Fluc and Gluc systems after reconstitution of influenza A virus polymerase complex**. (**A**) Polymerase activity assayed by a viral UTR-driven Gluc reporter gene. (**B**) Detection and quantification of RNP activity by the Gluc or Fluc reporter systems after transfection. 293T cells were transfected with plasmids encoding the PB2, PB1, PA, and NP genes of A/Quail/HK/G1/97 (H9N2) plus reporter plasmids polI-Gluc or pYH-Fluc. Supernatants (cell-free culture medium) and cell lysates were assayed for luciferase activity using appropriate substrates by a luminometer. (**C**) Kinetics of G1 RNP activity assayed using the Gluc reporter system after virus infection. A549 cells were transfected with plasmid polI-Gluc and infected at 6 h post-transfection with influenza virus G1 at a MOI of 1. Luciferase activity in supernatants was assayed at 0, 3, 6, 9, 12, and 24 h post-G1 (H9N2) infection and expressed as RLU per well. Data are expressed as standard errors of the mean (SEM) from three independent experiments performed in triplicate.

A virus-inducible Fluc reporter gene system has been developed and used for quantification of influenza virus polymerase activity [11]. To assess the potential of *Gaussia *protein as a reporter, we constructed artificial RNA segments encoding the *Gaussia *protein under the control of the UTRs of influenza A/WSN/33 NP segment. These artificial RNA segments were cloned into the RNA polymerase I promoter/terminator cassette to produce RNA transcripts containing no additional nucleotide sequences or modifications at either the 5' or 3' end. The vector polI-Gluc and plasmids carrying the PB1, PB2, PA, and NP segments of influenza virus G1 (H9N2) were co-transfected into 293T cells, and RNP activity was determined. Around a 600-fold higher luciferase activity was detected in supernatants at 18 h post-transfection as compared with that in mock-transfected cells (Figure 1A). Furthermore, Gluc signal accumulated with time. In contrast, the Fluc reporter gene signal peaked at 24 h post-transfection and subsequently remained at this level (Figure 1B).

The efficacy of the Gluc reporter system in assessing viral infection was examined. Little luciferase protein expression was detected from the construct in the absence of an authentic influenza A virus polymerase, similar to luciferase signals of non-transfected cells (data not shown). FluA polI-Gluc-transfected A549 cells were infected with influenza virus G1 (MOI of 1), and supernatants were harvested for luciferase analysis at 0, 3, 6, 9, 12, and 24 h post-infection (p.i.; Figure 1C). Signal was detected as early as 6 h p.i. At 24 h p.i., luciferase activity in G1-infected A549 cell supernatants was ~20-fold higher than that in mock-treated cells (Figure 1C). Thus, the Gluc system was capable of quantifying RNP activity and performed similar to the Fluc reporter system.

### Sensitivity of Gluc reporter system

Gluc is > 1000-fold more sensitive than the American *firefly *or sea pansy *Renilla reniformis *luciferases [2]. To compare sensitivities of the Gluc *versus *Fluc reporter systems, we transfected 293T cells with six plasmids: the vectors polI-Gluc and pYH-Fluc and plasmids encoding the G1 (H9N2) viral RNP. Supernatants of Gluc-transfected cells showed a 50-fold higher bioluminescent activity compared with Fluc-transfected cells at 24 h posttransfection (Figure 1B), again indicating the sensitivity of the Gluc-based system.

### Thermostability of Gluc reporter system

**Figure 2:**
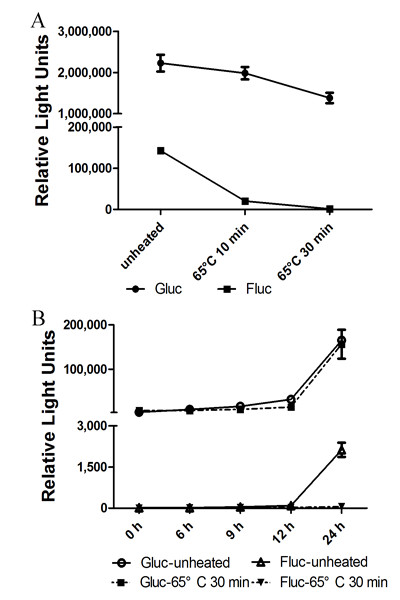
**Heat-sensitivity of Gluc and Fluc**. (**A**) RNP activity in polI-Gluc- and pYH-Fluc-transfected 293T cells after heat-treatment. 293T cells were transfected with plasmids encoding the PB2, PB1, PA, and NP segments of influenza G1 (H9N2) plus reporter plasmids polI-Gluc and pYH-Fluc. Supernatants and corresponding cell lysates were heated at 65°C for 10 or 30 min, and then Gluc and Fluc activities were assayed. (**B**) RNP activity in G1-infected A549 cells after heat treatment. Polymerase activity assays were performed in A549 cells transfected with vRNA *Gaussia *and *firefly *reporter plasmids and then infected with H9N2 at 6 h post-transfection. Supernatants and corresponding cell lysates were harvested for assay of Gluc and Fluc activity at appropriate times. Data are expressed as standard errors of the mean (SEM) from three independent experiments performed in triplicate.

Gluc is stable in culture medium (half-life around 6 d) [7], and therefore samples can be stored at 4°C for several days without any significant change in Gluc activity. Gluc is also heat stable at 65°C [15]. Influenza viruses are not heat-resistant and can be inactivated at 56°C for 30 min or 100°C for 1 min[16,17], and at 65°C for 30 min or 100°C for 2 min for H5N1 virus[18]. We compared Fluc and Gluc activities after heat treatment. Vectors polI-Gluc and pYH-Fluc were introduced together by both transfection and influenza virus infection and supernatants were harvested for Gluc assay and cells lysates for Fluc analysis. All samples were treated at 65°C for 10 or 30 min, and then Gluc and Fluc activities were determined. Gluc-transfected supernatants maintained > 90% activity after treatment at 65°C for 10 min and > 70.0% after 65°C for 30 min. In contrast, Fluc activity decreased to < 10% after heating for 10 min and was almost totally absent after 30 min (Figure 2A). Similar results were found by viral infection (Figure 2B).

### Determination of optimum infectious dose

**Figure 3:**
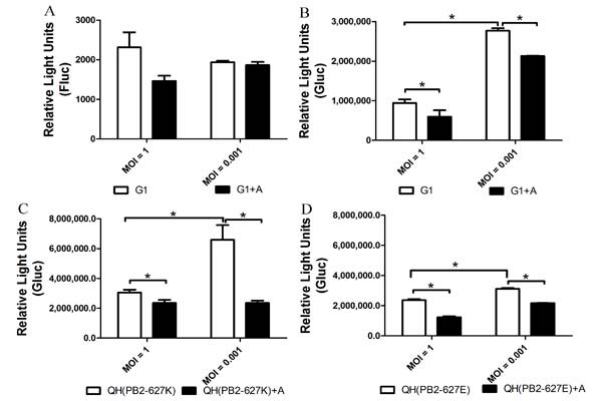
**The effect of virus dose and addition of antivirals on RNP activity**. A549 cells were transfected with plasmids pYH-Fluc (**A**) or polI-Gluc (**B**, **C**, and **D**) and incubated with or without amantadine (0.21 μM) for 30 min at 6 h post-transfection. Infections were performed with recombinant viruses G1 (**A**, **B**), QH (PB2-627K) (**C**), or QH (PB2-627E) (**D**) at a MOI of 1 and 0.001. At 24 h p.i., cells were lysed, and Fluc (**A**) and Gluc activity (**B**-**D**) were assayed. Data are expressed as standard errors of the mean (SEM) from three independent experiments performed in triplicate. A, amantadine; * *P *< 0.05.

Fluc reporter quantitation was linear in 293T cell infected with influenza virus at a MOI of 0.001-0.1 [11]. To elucidate the optimal infectious dose of the Gluc system, we transfected A549 cells (1 × 10^5^) with both pYH-Fluc and polI-Gluc, and infected cells with influenza A virus G1 at a MOI of 1 or 0.001, respectively. Fluc and Gluc activity was assayed following 24 h incubation at 37°C (Figure 3). Gluc (Figure 3B-D), but not Fluc (Figure 3A) activity was significantly different at a MOI of 0.001 as compared with a MOI of 1. Thus, our findings suggested that a MOI of 0.001 was better than that of 1 for the Gluc reporter assay under these conditions.

### Verification of PB2-K627E-induced RNP activity

**Figure 4:**
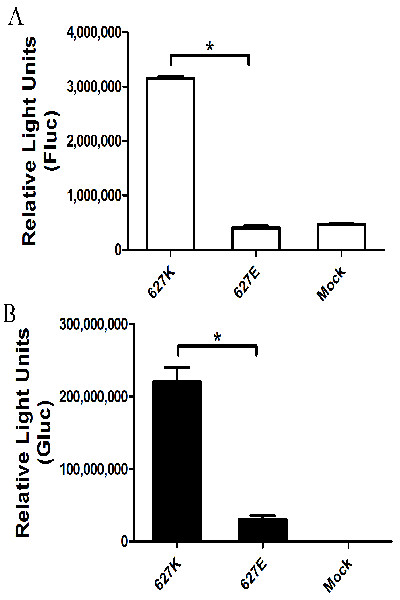
**RNP activity of QH virus containing PB2-627K and PB2-627E**. Fluc (**A**) and Gluc (signaling was detected after 1:10 dilution; **B**) were expressed from a synthetic viral RNA respectively when the influenza virus polymerase complex was present. 293T cells were transfected in 96- (Gluc; 5 × 10^4^/well) and 24-well (Fluc; 1 × 10^5^/well) plates. Control cells were mock transfected with the reporter plasmid alone. Data are expressed as standard errors of the mean (SEM) from three independent experiments performed in triplicate. * *P *< 0.05.

A single residue at position 627 in the PB2 subunit of the influenza polymerase (PB2-627) has been reported to be critical for polymerase activity in a species-specific fashion [19-23]. Most human influenza viruses possess a lysine at this position (PB2-627K), and most avian viruses possess glutamic acid (PB2-627E). PB2-627K is correlated with enhanced polymerase activity [19] and the high virulence [24] of H5N1 viruses in mice. PB2-627E attenuates viral replication efficiency and pathogenicity in mammals [25]. We verified these findings using the Gluc reporter system by both transfection with viral RNPs (Figure 4) and infection with QH (PB2-627K) and QH (PB2-627E) viruses (Figure 3C &3D). Signal was stronger in the Gluc than in the Fluc reporter system. A significant difference between PB2-627K and PB2-627E, which was consistent with other reports, was detected using the Gluc reporter system at a MOI of 0.001, but not at a MOI of 1 (Figure 3C &3D). Furthermore, this effect was blocked by addition of amantadine, an influenza virus entry inhibitor (Figure 3B-D; *P *< 0.05).

## Discussion

The influenza virus RNA-dependent RNA polymerase complex subunits (PB1, PB2, and PA) and NP catalyze both genome replication and transcription. This complex is also involved in influenza virus host adaptation [26]. In particular, a mutation in residue 627 of the PB2 segment (glutamic acid to lysine) is necessary for avian and swine influenza viruses to infect humans [27]. The H5N1 reassortant virus that contains a PB2 segment from human seasonal H3N2 is more virulent than the parental H5N1 strain in a mouse model [28]. Recently, a number of laboratories use Fluc reporter system to detect RNP activity. As for the limitations of Fluc system for RNP assay by virus infection, a system for determining influenza virus polymerase activity which would be more sensitive, easier to handle and allow more widespread use is essential.

Here, we first introduced *Gaussia *luciferase into a viral UTR-driven reporter system. *Gaussia *luciferase is a monomeric protein from the copepod marine organism *Gaussia princeps*. The small size of this gene (555 bp), together with its relative lack of toxicity, makes it suitable for vector construction and application. Moreover, *Gaussia *luciferase is naturally secreted and has a broad pH optimum as compared with other luciferases, with peak activity at pH 7.7 [29]. The Gluc reporter gene was activated and expressed via reconstitution of influenza A virus polymerase complex by either transfection or direct viral infection (Figure 1). The Gluc reporter produced a strong signal that accumulated with time (600- and 20-fold higher than controls in transfected and virus-infected supernatants, respectively; Figure 1), while Fluc signals saturated at 24 h p.i. with similar observation reported by Sun et al [30]. Furthermore, the Gluc reporter gene system was both much more sensitive and more heat stable than the Fluc system (Figs. 1B &2B).

Fluc was activated by G1 (H9N2) infection, but did not differ between one (MOI of 1) and multiple infection cycles (MOI of 0.001). Additionally, no significant effect of amantadine on G1 vRNP activity was found at neither MOI of 1 nor 0.001 (Figure 3A). Notably, stronger signal was detected at MOI of 0.001 than that of 1 for the Gluc reporter assay. Furthermore, signal produced by the Gluc system at MOI of 1 or 0.001 could be modified by addition of amantadine, an influenza virus ion blocker (Figure 3B-D), implying the powerful potency of Gluc system for RNP assay and the screening of antiviral compounds.

Similarly, significant differences between induction by PB2-627K and PB2-627E were detected by both the Gluc and Fluc reporter systems after reconstitution of viral RNPs by transfection (Figure 4), as well as after multiple infection cycles using the Gluc reporter system (Figure 3C-D). Our data suggest that multiple infection cycles might be better for the Gluc reporter system, which may be explained by the efficiency of infection at a low concentration of virus and the resultant amplification of the Gluc signal, showing that the effects of PB2-627K/E or amantadine on influenza vRNP activity were elucidated more effectively by the Gluc reporter system.

## Conclusion

Generally, Gluc system constructed here provided an alternative method for evaluating influenza virus polymerase activities, and showed much more advantages than traditional Fluc system. Firstly, Gluc luciferase was naturally secreted from cells into culture medium and could be detected readily. Secondly, much stronger signal intensity was generated by Gluc system. Finally, besides of its heat-resistance, Gluc reporter system works better during viral infection, which is suitable for RNP assay by direct virus infection and avoids constructing RNP plasmids. The reporter system based on secreted *Gaussia *luciferase activity for assaying influenza virus RNP functionality is sensitive, easier-detectable, and thermo-stable and is a potential tool for influenza virology study, especially for highly pathogenic avian influenza virus.

## Abbreviations

Gluc: *Gaussia *luciferase; Fluc: *firefly *luciferase; BSL-3: biosafety level 3 laboratory; vRNP: viral ribonucleoprotein; PB2: polymerase basic 2 protein; PB1: polymerase basic 1 protein; PA: polymerase acidic protein; NP: nucleoprotein; PCR: polymerase chain reaction; UTR: untranslated region; p.i.: post-infection; MOI: multiplicity of infection; VIRGS: virus-inducible reporter genes

## Competing interests

The authors declare that they have no competing interests.

## Authors' contributions

SYL and ZJF designed research. ZWF performed research. SYL contributed new reagents/analytic tools. ZWF and ZJF analyzed data and wrote the paper. QK, YZJ, ZY and TWH helped to construct partial plasmids. QK and DN helped to draft the manuscript. LLQ and WXB provided partial plasmids. All authors read and approved the final manuscript.
